# High Burden of Cardiac Disease in Pregnancy at a National Referral Hospital in Western Kenya

**DOI:** 10.5334/gh.404

**Published:** 2020-02-07

**Authors:** Rebecca Lumsden, Felix Barasa, Lawrence P. Park, Christian B. Ochieng, Joy M. Alera, Heather C. Millar, Gerald S. Bloomfield, Astrid Christoffersen-Deb

**Affiliations:** 1 Department of Medicine, Duke University Hospital, Durham, NC, US; 2 Department of Cardiology, Moi Teaching and Referral Hospital, Eldoret, KE; 3 Duke Global Health Institute, Duke University, Durham, NC, US; 4 LVCT Health, Nairobi, KE; 5 Directorate of Reproductive Health, Moi Teaching and Referral Hospital, Eldoret, KE; 6 Department of Obstetrics and Gynecology, University of Toronto, Toronto, CA; 7 Duke Clinical Research Institute, Duke University, Durham, NC, US; 8 Department of Obstetrics and Gynecology, University of British Columbia, Vancouver, CA

**Keywords:** Rheumatic heart disease (RHD), pregnancy, sub-Saharan Africa (SSA), Kenya, maternal mortality

## Abstract

**Background:**

Cardiac disease is a leading cause of non-obstetric maternal death worldwide, but little is known about its burden in sub-Saharan Africa.

**Objectives and Methods:**

We conducted a retrospective case-control study of pregnant women admitted to a national referral hospital in western Kenya between 2011–2016. Its purpose was to define the burden and spectrum of cardiac disease in pregnancy and assess the utility of the CARPREG I and modified WHO (mWHO) clinical risk prediction tools in this population.

**Results:**

Of the 97 cases of cardiac disease in pregnancy, rheumatic heart disease (RHD) was the most common cause (75%), with over half complicated by severe mitral stenosis or pulmonary hypertension. Despite high rates of severe disease and nearly universal antenatal care, late diagnosis of cardiac disease was common, with one third (38%) of all cases newly diagnosed after 28 weeks gestational age and 17% diagnosed after delivery. Maternal mortality was 10-fold higher among cases than controls. Cases had significantly more cardiac (56% vs. 0.4%) and neonatal adverse events (61% vs. 27%) compared to controls (p < 0.001). Observed rates of adverse cardiac events were higher than predicted by both CARPREG I and mWHO risk scores, with high cardiac event rates despite low or intermediate risk scores.

**Conclusions:**

Cardiac disease is associated with significant maternal and neonatal morbidity and mortality among pregnant women in western Kenya. Existing clinical tools used to predict risk underestimate adverse cardiac events in pregnancy and may be of limited utility given the unique spectrum and severity of disease in this population.

## Introduction

Cardiac disease is a leading cause of maternal mortality worldwide, contributing to over 10% of all maternal mortality cases globally [4,7,10]. However, the epidemiology and outcomes associated with cardiac disease in pregnancy vary significantly between high income (HIC) and low- and middle-income countries (LMICs). Unlike in HICs where the burden of cardiac disease is primarily congenital heart disease, rheumatic heart disease (RHD) remains common in LMICs [6,15,19,20].

Sub-Saharan Africa (SSA) bears a disproportionately high burden of RHD among women of reproductive age, yet the prevalence of cardiac disease in pregnancy in this region is largely unknown [22,30]. Data from the Global Rheumatic Heart Disease Registry (REMEDY), a multinational registry including over 3000 patients with RHD from 12 African countries, found that nearly two-thirds of all cases were women of reproductive age. However, contraceptive use and access to both reliable and specialized antenatal care remain low across the region, making this a particularly high-risk population for adverse outcomes in pregnancy [22,29,30].

Maternal mortality rates in Kenya have been slow to improve despite the provision of free maternity services by the Ministry of Health [26]. Persistently high rates of maternal mortality are in part due to the increasing proportion of women presenting with complex medical conditions at the time of delivery [24]. A maternal mortality review from a large referral hospital in western Kenya found that one-third of all maternal deaths were attributable to non-obstetric causes, including cardiovascular disease [28]. Concurrently, a study from the outpatient cardiology clinic of the same hospital revealed that over half of all patients attending were women of reproductive age with RHD [5]. Despite the known morbidity from cardiac disease in pregnancy, few data exist to quantify the burden and types of cardiac disease in pregnancy in Kenya.

To address this gap in knowledge, we sought to describe the spectrum of cardiac disease in pregnancy and associated maternal and neonatal outcomes using medical record data from a high-volume tertiary care hospital in western Kenya. Additionally, we aimed to evaluate the utility of validated risk indexes—the Cardiac Disease in Pregnancy (CARPREG I) and modified World Health Organization (mWHO) scores—in assessing risk of adverse cardiac events within this population [14,20]. Our overall objective was to better understand the impact of cardiac disease in pregnancy, in order to guide efforts to improve maternal health in this region.

## Methods

### Study Site

Moi Teaching and Referral Hospital (MTRH) is one of only two national referral hospitals in Kenya. The standalone maternity hospital at MTRH performs over 12,000 deliveries annually. Cardiovascular care and research are organized through the infrastructure of a National Heart, Lung and Blood Institute (NHLBI)-funded Center of Excellence [1]. Ethical approval for the study was obtained from both the Institutional Review and Ethics Committee (IREC) at the Moi University School of Medicine and the University of Toronto.

### Study Design

This was a retrospective case-control study of pregnant women hospitalized at MTRH between January 2011 and April 2016. Cases included all women with a known cardiac diagnosis prior to or during the pregnancy period who were admitted to the hospital for any reason during pregnancy, or up to 6 weeks postpartum. Cases were identified using ICD-9 and ICD-10 codes from inpatient admissions, echocardiogram orders and pharmacy records. Cases were matched to pregnant controls without any known history of cardiac disease based on age (+/– 2 years) and parity (+/– 2). Controls were excluded if insufficient data were able to be located from the medical record.

Medical charts were reviewed by two data collectors (JA, RL). Information pertaining to the incident pregnancy and cardiac disease were obtained from provider notes, standardized labor and delivery forms, and transthoracic echocardiogram (TTE) and electrocardiogram (ECG) reports. All cardiac diagnoses listed during the clinical encounter were recorded. Objective data from the first medical encounter during pregnancy were collected if present, including: blood pressure, heart rate, oxygen saturation, New York Heart Association (NYHA) class of heart failure, and TTE and ECG findings. Left ventricular ejection fraction (LVEF), right ventricular systolic pressure (RVSP), and type and severity of valvular lesions were obtained from hospital TTE reports. Left ventricular ejection fraction (LVEF) was recorded as reported using the following categories: normal (>55%), mild dysfunction (45–55%), moderate dysfunction (30–44%), or severe dysfunction (<30%). All ECGs were interpreted by a single cardiologist (FB). Cases with discrepancies between diagnoses were reviewed by two cardiologists (FB, GSB). Those without objective cardiac disease were excluded from the study.

We generated a standardized definition of RHD based on adapted criteria from the World Heart Federation and data available from TTE reports [9]. We defined RHD as: any mitral stenosis (MS) or aortic stenosis (AS); presence of both mitral regurgitation (MR) and aortic regurgitation (AR); or isolated MR or AR, if moderate or severe. All cases under 20 years old or with equivocal lesions were individually reviewed by two cardiologists (FB, GSB) to determine inclusion using World Heart Federation criteria for borderline RHD [9].

Our primary outcome was maternal death during pregnancy or up to 6 weeks postpartum. Secondary outcomes included: cardiac adverse events (cardiac arrest, new arrhythmia, heart failure, myocardial infarction, stroke, endocarditis, or admission to the cardiac intensive care unit [CCU]), obstetric adverse events (Cesarean-section, vacuum-assisted delivery, induction of labor, postpartum hemorrhage, pre-eclampsia/eclampsia, or venous thrombosis event), or neonatal adverse events (intrauterine fetal demise (IUFD, fetal death ≥28 weeks gestational age). Other secondary outcomes included: neonatal death (<28 days after live birth), preterm delivery (<38 weeks gestational age), low birth weight (<2500 grams), admission to the intensive newborn unit, or APGAR score <7 at 1, 5 or 10 minutes).

### Statistical Analysis

A minimum of 145 participants were required in each arm to achieve 80% power in detecting a difference in the primary outcome, using the maternal mortality rate at MTRH of 0.3% and an estimated rate of 6% among cases based on literature [2,3,16,23]. Controls were matched to cases (2:1) in order to detect differences in secondary maternal and neonatal outcomes. Missing data points for objective measures pertaining to the incident pregnancy were dropped, whereas missing historical medical diagnoses were assumed to be “no.” Univariate analyses were performed using the Kruskal-Wallis test and Fisher’s exact test for continuous and categorical variables respectively.

We applied both the CARPREG I score and mWHO classification to estimate the risk of adverse events in pregnancy [8,13,20]. A CARPREG I score of 0–4 was generated for each case, with one point each for: prior cardiac event (history of heart failure, stroke, or arrhythmia before pregnancy) NYHA class III or IV or oxygen saturation <80% at presentation; left ventricular inflow and outflow obstruction (mitral valve area (MVA) < 1.5 cm^2^ or severe aortic stenosis); or reduced left ventricular systolic function (LVEF < 45% in our sample) [8]. We adapted the mWHO classification of maternal cardiovascular risk (class I–IV), as outlined in the European Society of Cardiology guidelines, as follows: [6,23] mWHO class I included repaired simple, congenital lesions; class II included all unrepaired congenital cases and any arrhythmia on ECG; class III included mild LVEF impairment (LVEF < 45%) and any valvular heart disease not otherwise considered class I or IV. Class IV included pulmonary hypertension of any cause (RVSP by echocardiogram > 35 mmHg), severe systemic ventricular dysfunction (LVEF < 30% or NYHA class III or IV), previous peripartum cardiomyopathy with any residual LV impairment, severe mitral stenosis, or severe, symptomatic aortic stenosis. Cases were categorized into the higher class if multiple features were present. Logistic regression was performed to calculate the observed risk of cardiac and neonatal outcomes based on number of predictor variables for both CARPREG I and mWHO scores, independently. Analyses were carried out using STATA (Version 15, College Station, TX: Stata Corporation).

## Results

### Demographics

A total of 97 cardiac cases in pregnancy were identified and matched to 242 controls. Median age of cardiac cases was 26 years compared to 28 for controls (p = 0.007, Table 1). There was no difference in gravidity or parity between groups. Nearly two-thirds (63.9%) of cardiac cases had a previous delivery, while 30% had three or more. Almost all participants had at least one routine antenatal visit. There was no significant difference in rates of comorbid conditions between groups, including hypertension, diabetes, HIV, pulmonary disease or renal disease (9.3% vs. 4.6%, p = 0.128).

**Table 1:** Baseline characteristics at time of presentation to care.

	Cardiac Cases (n = 97)	Controls (n = 242)	p-value

**Age, median (range)**	26 (16–50)	28 (18–41)	0.0069
**Gravidity, median (range)**	2 (1–8)	2 (1–6)	0.1505
**Parity, median (range)**	1 (0–8)	1 (0–5)	0.1094
Nulliparous, n (%)	35 (36.1)	107 (44.2)	
Parity 1–2, n (%)	33 (34.0)	86 (35.5)	
Parity ≥ 3, n (%)	29 (29.9)	49 (20.3)	
**Received antenatal care, n (%)**^1^	73 (97.3)	214 (97.3)	1.000
**History of non-obstetric, comorbid medical condition during pregnancy, n (%)**^2^	11 (4.6)	9 (9.3)	0.124
**Obstetric high-risk, n (%)**^3^	39 (40.2)	75 (31.0)	0.127

^1^ Had one or more antenatal visit. Antenatal care unknown for 22 cardiac cases (n = 75), 22 control cases (n = 220). ^2^ Includes: hypertension, diabetes mellitus, HIV, pulmonary disease, renal disease, thyroid disease. ^3^ Includes: previous preterm birth, previous C-section, previous IUFD, multiple gestations (current pregnancy), or seen in high-risk antenatal clinic.

### Clinical profile of cardiac disease in pregnancy

Acquired causes of cardiac disease were present in 90 of 97 cases (92.3%), while electrical and congenital causes were less common (15.5% and 7.2% respectively, Table 2). Most cases (75.3%) had RHD, of which half had mitral stenosis, and one-third of those were severe. Pulmonary hypertension, defined as RVSP ≥ 35 mmHg on TTE, was present in 61% of cases, with a median RVSP of 64mmHg (IQR 39–120). Only 10% of cases had an LVEF < 45%. Of the 57 cases with NYHA class recorded at the initial encounter, 35 (61.4%) were class III or IV.

**Table 2:** Clinical Profile of Cardiac Disease Cases in Pregnancy at MTRH.

Type of Cardiac Disease, n (%)	Cases (n = 97)

**Acquired**	90 (92.8)
RHD	73 (75.3)
Cardiomyopathy^1^	4 (4.1)
Primary pulmonary hypertension	15 (15.5)
**Congenital**	7 (7.2)
**Electrical/Arrhythmia**	15 (15.5)
**Valvular Lesions**^2^	
Mitral Regurgitation	45 (63.4)
Mitral Stenosis	40 (56.3)
Severe	22 (31.0)
Moderate	8 (11.3)
Mild	6 (8.5)
Aortic regurgitation	24 (33.8)
Aortic stenosis	4 (5.6)
**Severity of Disease and Symptoms at First Admission**	

NYHA Class at presentation^3^	
I or II	22 (38.6)
III or IV	35 (61.4)
Systolic BP, median (range)	114 (80–180)
Diastolic BP, median (range)	70 (40–122)
HR, median (range), n = 96	92 (56–205)
Oxygen saturation, median (range)	92 (56–99)
LVEF < 45%, n (%)^4^	9 (9.8)
Right Ventricular Systolic Pressure, mmHg, median (IQR)	64 (39–120)
RVSP ≥ 35 mmHg, n (%)	57 (61.3)
RVSP < 35 mmHg, n (%)	36 (38.7)
**Timing of Cardiac Diagnosis, n (%)**^5^	

Before Pregnancy	50 (53.8)
During Pregnancy	22 (23.7)
1^st^ Trimester (GA < 14 weeks)	1 (1.1)
2^nd^ Trimester (GA 14–27.6 weeks)	5 (5.4)
3^rd^ Trimester (GA > 28 weeks)	16 (17.2)
Intra-partum (labor – <24-hrs post-delivery)	5 (5.4)
Postpartum (≥24-hrs – 6 weeks post-delivery)	16 (17.2)
**Seen by any cardiologist prior to this pregnancy, n (%)**^6^	48 (52.2)
**Cardiac Event before Pregnancy, n (%)**	10 (10.3)
Heart Failure	9 (9.3)
Arrhythmia	2 (2.1)
Stroke/TIA or MI	0 (0)
**History of cardiac surgery, n (%)**	10 (10.4)
Valve repair	2 (2.1)
Valve replacement	7 (7.3)

^1^ Includes: peripartum or dilated cardiomyopathy. ^2^ TTE data missing for 2 RHD cases (n = 71). ^3^ Unknown for 40 cases (n = 57). ^4^ Unknown for 5 cases (n = 92). ^5^ Unknown for 4 cases (n = 93). ^6^ Unknown for 5 cases (n = 92).

Only half of cases (53.8%) were diagnosed with cardiac disease prior to the incident pregnancy (Table 2). Of the 43 cases diagnosed during pregnancy, the majority (n = 37, 86.0%) were diagnosed late in the 3^rd^ trimester, or after delivery.

### Outcomes

The maternal mortality rate among cardiac cases was 9.3%, with no deaths in the control group (p = 0.001, Table 3). Half of all maternal deaths had severe mitral stenosis (n = 4) and nearly all had pulmonary hypertension (n = 7). Despite the severity of disease among these cases, four of the nine were undiagnosed prior to the incident pregnancy. Six of the nine maternal deaths happened in the postpartum period, with four occurring more than 14 days after delivery (Table 4).

**Table 3:** Adverse cardiac, obstetric and neonatal events during pregnancy.

Variable	Cardiac Cases	Controls	P-value

**Maternal mortality, n (%)**	9 (9.3)	0 (0)	<0.001
**Any Adverse Event**^1^	71 (79.8)	54 (25.1)	<0.001
**Any Cardiac Event, n (%)**	54 (55.6)	1 (0.4)	<0.001
Cardiac arrest	3 (3.1)	0 (0)	0.023
Arrhythmia	7 (7.2)	0 (0)	<0.001
Congestive Heart Failure	41 (42.3)	0 (0)	<0.001
Stroke	2 (2.1)	0 (0)	0.081
CCU admission	21 (21.7)	1 (0.4)	<0.001
**Any Obstetric Event, n (%)**^2^	63 (72.4)	73 (30.8)	<0.001
Cesarean-section	14 (15.4)	42 (17.6)	0.743
Vacuum-assisted delivery	18 (19.8)	0 (0)	<0.001
Induction of labor	30 (37.0)	24 (10.3)	<0.001
Postpartum hemorrhage	5 (5.2)	5 (2.1)	0.157
Pre-eclampsia/eclampsia	14 (14.4)	13 (5.4)	0.008
Venous thromboembolism	10 (10.3)	1 (0.4)	0.000
**Any Neonatal Event, n (%)**	49 (61.3)	58 (27)	<0.001
Intrauterine fetal demise	10 (11.2)	7 (3.0)	0.009
Neonatal death	4 (5.1)	2 (0.9)	0.039
Preterm delivery	32 (41.6)	23 (10.8)	<0.001
Low birth weight	24 (30.4)	18 (7.8)	<0.001
Newborn Unit admission	16 (23.8)	23 (10.2)	0.007
APGARS < 7 (at 1, 5, or 10 min)	6 (9.7)	11 (5.0)	0.223
**Timing of first hospitalization**			
Antenatal, n (%)^3^	60 (67.4)	5 (2.1)	<0.001
GA in weeks, median (range)	33.2 (6.3–39.5)	35.1 (14.1–37.4)	
Intrapartum, n (%)	28 (29.8)	230 (95.0)	<0.001
Postpartum, n (%)	9 (9.3)	7 (2.9)	0.020
Days postpartum, median (range)	12 (2–27)	1 (0–21)	

^1^ Any maternal death, cardiac or neonatal adverse event. ^2^ Unknown for 10 cases and 10 controls (n = 87 cases, n = 237 controls). ^3^ Unknown for 8 cardiac cases (n = 89).

**Table 4:** Description of all maternal deaths.

Time of Death	Gravida Parity	Cardiac Disease	Time of Cardiac Diagnosis	NYHA Class	ECHO Characteristics	ECG	Method of Delivery	Pregnancy outcome

**Antenatal** – 27.4 weeks	G2P1	RHD	Before Pregnancy	IV	LVEF > 55% Severe MS RVSP 71 mmHg	NSR	N/A	Miscarriage
**Intrapartum**	G1P0	Pulmonary Hypertension	During pregnancy – 3^rd^ trimester	Unknown	LVEF > 55% RVSP 41 mmHg	NSR	Induction of labor	Live birth
**Intrapartum**	G2P1	RHD	During pregnancy – 3^rd^ trimester	III	LVEF > 55% RVSP 92 mmHg	NSR	Induction of labor	IUFD
**Postpartum** – 2 hours	G5P4	RHD	During pregnancy – 1^st^ trimester	IV	LVEF 30–45% Severe MS RVSP 77 mmHg	A. fib.	N/A	Miscarriage
**Postpartum** – 13 hours	G2P0	RHD	Before Pregnancy	III	LVEF 30–45% Severe MS RVSP 69 mmHg	A. fib.	Emergent C-section	IUFD
**Postpartum** – day 17	G4P3	RHD	Unknown	Unknown	LVEF 45–55% Severe MS RVSP 140 mmHg	NSR	Spontaneous vaginal delivery	Live birth
**Postpartum** – day 26	G1P0	PPCM	Postpartum	Unknown	LVEF <30% RVSP 53 mmHg pericardial effusion, LV apical thrombus	STEMI	Emergent C-section	IUFD
**Postpartum** – day 31	G2P2	Congenital	Before Pregnancy	IV	EF 30–45% RVSP 164 mmHg	NSR	Unknown	Unknown
**Postpartum** – day 58	G1P0	RHD	During pregnancy – 2^nd^ trimester	Unknown	LVEF > 55%	NSR	Induction of labor	Neonatal death

Abbreviations: RHD = rheumatic heart disease; LVEF = left ventricular ejection fraction; RVSP = right ventricular systolic pressure; PPCM = peripartum cardiomyopathy; IUFD = intrauterine fetal demise; NSR = normal sinus rhythm; A fib = atrial fibrillation; STEMI = ST-elevation myocardial infarction; N/A = Not applicable.

Nearly all cardiac cases (79.8%) had at least one cardiac, obstetric, or neonatal adverse event related to pregnancy, as compared to only 25% of controls (p < 0.001, Table 3). Congestive heart failure was the most common cardiac event, occurring in 42.3% of cases, followed by new arrythmia in 7%, and 2% had a stroke. Nearly one-quarter (21.7%) of all cases were admitted to the cardiac ICU at least once during the pregnancy period. Significantly higher rates of neonatal events were seen among cardiac cases as compared to controls, including: intrauterine fetal demise (11.2% vs 3.0%, p = 0.009), low birth weight (30.4% vs 7.8%, p < 0.001), and preterm delivery (41.6% vs 10.8%, p < 0.001). Cases had higher rates of induction of labor (37.0% vs. 10.3%, p < 0.001) and vacuum-assisted deliveries (19.8% vs. 0%, p < 0.001) compared to controls, but there was no difference in C-section rates between groups.

### Prediction Scores

**Figure 1:**
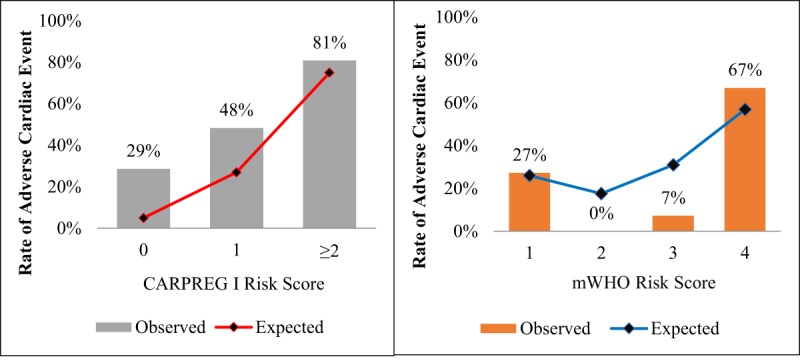
**Observed vs. Expected Cardiac Event Rates based on CARPREG I and mWHO Scores.** Observed rates of adverse cardiac events were higher than predicted by the CARPREG I score with expected rates of 5%, 27 and 75% for scores 0, 1 and ≥2), whereas rates of adverse cardiac events were more closely predicted by the mWHO score for caes in low mWHO class (I) and high mWHO (IV) classes (9.9% vs. 50.3%, respectively).

**Figure 2:**
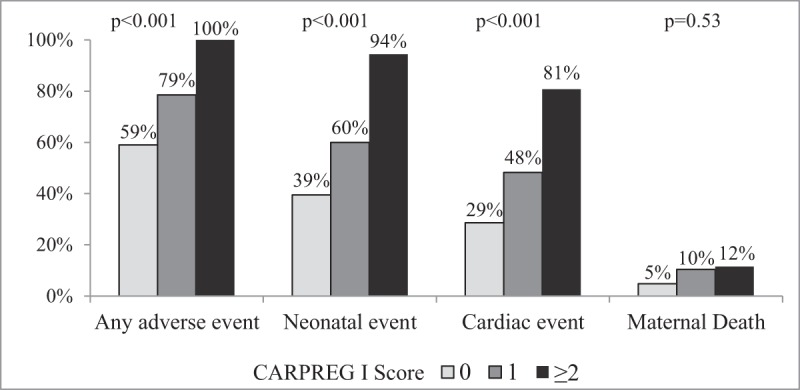
**Observed Rates of Adverse Events based on CARPREG I Score.** High rates of adverse cardiac and neonatal events were observed despite low and intermediate CARPREG I scores (0 or 1), while a high score (≥2) was associated with nearly universal rates of adverse events.

Observed rates of adverse events were higher than predicted by the CARPREG I and mWHO risk scores. Cardiac events occurred in 29%, 48%, and 81% of cases with CARPREG I scores of 0, 1, and ≥2 respectively, as compared to expected rates of 5%, 27%, and 75% (Figure 1) [13]. Among those with a CARPREG I score of 0, 59% had some adverse event (cardiac, obstetric or neonatal Figure 2). A CARPREG I score ≥2 carried a 100% rate of any adverse event.

Similarly, high rates of adverse events occurred among cases with low mWHO class. Of those with mWHO class I, 27% experienced an adverse cardiac event (Figure 1). Observed rates of cardiac events in these groups were similar to predicted from existing data in emerging countries for mWHO class I (27% vs. 26%) and class IV (67% vs. 57%) (20). However, most cases were classified as either mWHO class I (23%) or mWHO class IV (68%), with very few cases classified as class II or III (1% and 7%, Table 5).

**Table 5:** Comparison of Adverse Events using CARPREG I and mWHO Risk Scores.

	N (%)	Cardiac Event, n (%)	Neonatal Event, n (%)^	Maternal Death, n (%)

**CARPREG I score**	97	47 (48.5)	47 (58.0)	8 (8.3)
**0**	42 (43.3)	12 (28.6)	15 (39.5)	2 (4.8)
**1**	29 (29.9)	14 (48.3)	15 (60.0)	3 (10.3)
**2**	22 (22.7)	17 (77.3)	14 (93.3)	1 (4.6)
**3**	4 (4.1)	4 (100)	3 (100)	2 (50.0)
**mWHO class**	95*	47 (49.5)	46 (58.2)	8 (8.4)
**I**	22 (23.2)	6 (27.3)	10 (47.6)	2 (9.1)
**II**	1 (1.1)	0 (0)	0 (0)	0 (0)
**III**	7 (7.4)	1 (14.3)	2 (33.3)	0 (0)
**IV**	65 (68.4)	40 (61.5)	34 (66.7)	6 (9.2)

^ Neonatal data only available for n = 81 cases. * Unable to calculate mWHO scores for 2 cases due to missing data.

## Discussion

This study is one of the first to investigate the burden of cardiac disease in pregnancy in Kenya. Maternal mortality was nearly ten-fold higher among pregnant women with cardiac disease hospitalized at a national, referral hospital in western Kenya compared to women without cardiac disease over a five-year period. Rheumatic heart disease was the most common cause of cardiac disease and was often complicated by severe mitral stenosis or pulmonary hypertension. Observed rates of adverse cardiac and neonatal events were higher than predicted using existing CARPREG I and mWHO risk models.

The predominance of complicated RHD seen among pregnant women in our study mirrors the disproportionate burden of RHD disease seen among women of reproductive age in SSA, where RHD remains endemic [5,22,29]. Data from the Registry of Pregnancy and Cardiac Disease (ROPAC), the largest registry of pregnant women with cardiac disease globally, illustrates that 55% of women enrolled from LMICs had valvular heart disease, predominantly rheumatic mitral stenosis, and studies from South Africa estimate that RHD accounts 71–84% of all cases of antenatal heart disease [16,18,21,23]. Additionally, more than half of our cases had severe mitral stenosis and/or pulmonary hypertension, both of which can be contraindications to pregnancy [8,11,17]. Most had decompensated heart failure with NYHA class III or IV symptoms at initial presentation, a factor independently associated with increased risk of complications in pregnancy [16]. However, advanced, symptomatic cardiac disease was too often undiagnosed until late pregnancy, despite high attendance to routine antenatal care, suggesting huge gaps in screening and diagnosis of cardiac disease within routine, antenatal care in this highly endemic setting.

### Outcomes

Maternal mortality among cardiac cases was nearly 10-times higher than all-cause mortality among pregnant women in Kenya. With up to one-third of maternal deaths in western Kenya attributable to non-obstetric causes, our findings indicate that cardiac disease may be a significant, under-recognized threat to persistent maternal mortality in this region [25,26,27,28]. Notably, half of maternal deaths occurred in the late postpartum period, greater than 14 days after delivery, suggesting that the risk of significant adverse events extends beyond the traditional timeframe of routine hospitalization following obstetric delivery. Similar results were found in a prospective cohort of South African women with cardiac disease, where the highest rates of maternal death occurred between 44–150 days postpartum [16]. Thus, close postpartum monitoring and follow up may be as critical as early antenatal care in this population.

### Risk Prediction

Understanding and quantifying risk of cardiac disease in pregnancy is critical to pregnancy planning and early management strategies, especially in low-resource settings where surgical or other invasive interventions for cardiac disease are limited, but existing tools are inadequate. We found that the CARPREG I and mWHO risk prediction tools underestimated adverse events in our population, with high rates of cardiac events despite low CARPREG I and mWHO risk scores. Previous attempts to validate the CARPREG I score in other LMICs have demonstrated both over- and underestimation of risk of adverse events, likely due to the unique spectrum of disease specific to LMICs as compared to the North American cohort from which the CARPREG I score was derived [12,14]. Over half of our cases had pulmonary hypertension, a relative contraindication to pregnancy with significant maternal and neonatal risk, which is not captured in the CARPREG I score, and thus could account for underestimate of risk [8,14,17]. The mWHO risk score, which includes more disease-specific cardiac lesions, is still regionally limited. Sub-analysis from the ROPAC registry reveals that the mWHO risk score was still less effective in predicting cardiac events among women in LMICs compared to high income countries, and was particularly poor for women with acquired heart disease in LMICs [20].

Both scores fail to capture unique barriers present in low-resource settings that may be contributing to poor outcomes, including late presentation to routine, antenatal care, lack of adequate diagnostic technologies, and limited access to coordinated, subspecialized care [12,20]. While the severity of the underlying cardiac lesions remains the major driver of poor outcomes in pregnancy, late presentation to care and delayed diagnosis likely exacerbate the risk of maternal and neonatal complications in our population. Nearly all of our cases attended an antenatal clinic, and most had at least one prior pregnancy; yet, over one-third of cases were first diagnosed with cardiac disease after the second trimester, suggesting that identification of cardiac disease within routine antenatal care is low.

The CARPREG II score was recently derived from and validated in a Canadian population and found that late pregnancy assessment and lack of cardiac intervention prior to pregnancy were independent risk factors for cardiac event in pregnancy [12]. This newer score now includes these as weighted, predictor variables, suggesting that poor access and late presentation to antenatal care, on top of disease type and severity, are significant risk factors to adverse cardiac events in pregnancy in Kenya. Therefore, clinical strategies for earlier detection of cardiac disease during routine antenatal care, strengthened postpartum follow-up, and improved risk prediction scores are needed in the sub-Saharan population.

### Limitations

Overall, we found fewer cardiac cases than we anticipated based on the known burden of cardiac disease in the region. This may be due to the retrospective study design, which resulted in a smaller sample than expected in which to detect a statistical difference in the primary outcome between groups. Despite this, our data highlights the high absolute maternal death rate among women with cardiac disease. Given that we included only women hospitalized at a tertiary care facility, selection bias may contribute to overestimation of adverse events, and may affect the application of risk prediction models. Inconsistent record keeping from paper medical charts resulted in the potential for missing data, which we attempted to correct for by imputing historical medical data. However, it could have led to underestimation of outcomes among the control group. Similarly, this likely contributed to small subgroup sizes in risk prediction modeling (ex: few cases of mWHO class II and III) and limited use of the newer CARPREG II score in this population. Lastly, there were statistical differences between cardiac and non-cardiac cases with regards to both age and parity due to our phased approach to matching. However, these small statistical differences are unlikely to have clinically meaningful influences on our results. While these limitations suggest more robust, prospective studies need to be conducted to better guide our clinical management of these high-risk patients, this study provides critical information as one of the first to attempt to characterize the impact of cardiac disease in pregnancy in East Africa.

## Conclusions

Rheumatic heart disease remains the most common cause of cardiac disease seen in pregnancy, frequently complicated by advanced mitral stenosis and pulmonary hypertension. It is often diagnosed late in pregnancy, despite high rates of routine antenatal care. The existing CARPREG I and mWHO risk scores are limited tools to accurately assess risk in this population given the unique spectrum and severity of disease in this sub-Saharan population. Further prospective studies are needed to develop new risk prediction tools and enhanced strategies for early disease recognition within routine antenatal care practices in sub-Saharan Africa in order to improve maternal and neonatal outcomes in this high-risk population.

## References

[1] Binanay CA, Akwanalo CO, Aruasa W, Barasa FA, Corey GR, Crowe S, et al. Building sustainable capacity for cardiovascular care at a public hospital in western Kenya. J Am Coll Cardiol 2015; 66(22): 2550–60. DOI: 10.1016/j.jacc.2015.09.086 26653630 PMC4680855

[2] Cole TO, Adeleye JA. Rheumatic heart disease and pregnancy in Nigerian women. Clin Cardiol 1982; 5(4): 280–5. DOI: 10.1002/clc.4960050403 7083651

[3] Diao M, Kane A, Ndiaye MB, Mbaye A, Bodian M, Dia MM, et al. Pregnancy in women with heart disease in sub-Saharan Africa. Arch Cardiovasc Dis 2011; 104(6–7): 370–4. DOI: 10.1016/j.acvd.2011.04.001 21798468

[4] Kassebaum NJ, Barber RM, Bhutta ZA, Dandona L, Gething PW, Hay SI, et al. Global, regional, and national levels of maternal mortality, 1990–2015: a systematic analysis for the Global Burden of Disease Study 2015. The Lancet 2016; 388(10053): 1775–812. DOI: 10.1016/S0140-6736(16)31470-2 PMC5224694 27733286

[5] Lumsden RH, Akwanalo C, Chepkwony S, Kithei A, Omollo V, Holland TL, et al. Clinical and geographic patterns of rheumatic heart disease in outpatients attending cardiology clinic in western Kenya. Int J Cardiol 2016; 223: 228–35. DOI: 10.1016/j.ijcard.2016.08.069 27541662

[6] Mocumbi AO, Sliwa K, Soma-Pillay P. Medical disease as a cause of maternal mortality: the pre-imminence of cardiovascular pathology. Cardiovasc J Afr 2016; 27(2): 84–8. DOI: 10.5830/CVJA-2016-018 27213855 PMC4928173

[7] Regitz-Zagrosek V, Blomstrom Lundqvist C, Borghi C, Cifkova R, Ferreira R, Foidart JM, et al. ESC Guidelines on the management of cardiovascular diseases during pregnancy: the Task Force on the Management of Cardiovascular Diseases during Pregnancy of the European Society of Cardiology (ESC). Eur Heart J 2011; 32(24): 3147–97. DOI: 10.1093/eurheartj/ehr218 21873418

[8] Regitz-Zagrosek V, Roos-Hesselink JW, Bauersachs J, Blomstrom-Lundqvist C, Cifkova R, De Bonis M, et al. 2018 ESC Guidelines for the management of cardiovascular diseases during pregnancy. Eur Heart J 2018; 39(34): 3165–241. DOI: 10.1093/eurheartj/ehy340 30165544

[9] Remenyi B, Wilson N, Steer A, Ferreira B, Kado J, Kumar K, et al. World Heart Federation criteria for echocardiographic diagnosis of rheumatic heart disease—an evidence-based guideline. Nat Rev Cardiol 2012; 9(5): 297–309. DOI: 10.1038/nrcardio.2012.7 22371105 PMC5523449

[10] Say L, Chou D, Gemmill A, Tuncalp O, Moller AB, Daniels J, et al. Global causes of maternal death: a WHO systematic analysis. The Lancet Global health 2014; 2(6): e323–33. DOI: 10.1016/S2214-109X(14)70227-X 25103301

[11] Silversides CK, Colman JM, Sermer M, Siu SC. Cardiac risk in pregnant women with rheumatic mitral stenosis. The American Journal of Cardiology 2003; 91(11): 1382–5. DOI: 10.1016/S0002-9149(03)00339-4 12767443

[12] Silversides CK, Grewal J, Mason J, Sermer M, Kiess M, Rychel V, et al. Pregnancy outcomes in women with heart disease: the CARPREG II study. J Am Coll Cardiol 2018; 71(21): 2419–30. DOI: 10.1016/j.jacc.2018.02.076 29793631

[13] Siu SC, Sermer M, Colman JM, Alvarez AN, Mercier LA, Morton BC, et al. Prospective multicenter study of pregnancy outcomes in women with heart disease. Circulation 2001; 104(5): 515–21. DOI: 10.1161/hc3001.093437 11479246

[14] Siu SC, Sermer M, Harrison DA, Grigoriadis E, Liu G, Sorensen S, et al. Risk and predictors for pregnancy-related complications in women with heart disease. Circulation 1997; 96(9): 2789–94. DOI: 10.1161/01.CIR.96.9.2789 9386139

[15] Sliwa K, Böhm M. Incidence and prevalence of pregnancy-related heart disease. Cardiovasc Res 2014; 101(4): 554–60. DOI: 10.1093/cvr/cvu012 24459193

[16] Sliwa K, Libhaber E, Elliott C, Momberg Z, Osman A, Zuhlke L, et al. Spectrum of cardiac disease in maternity in a low-resource cohort in South Africa. Heart 2014; 100(24): 1967–74. DOI: 10.1136/heartjnl-2014-306199 25227705 PMC4251204

[17] Sliwa K, Van Hagen I, Budts W, Swan L, Sinagra G, Vazquez Blanco M, et al. Pulmonary hypertension and pregnancy outcomes: data from the Registry Of Pregnancy and Cardiac Disease (ROPAC) of the European Society of Cardiology. 2016 DOI: 10.1002/ejhf.594 27384461

[18] Sliwa K, Wilkinson D, Hansen C, Ntyintyane L, Tibazarwa K, Becker A, et al. Spectrum of heart disease and risk factors in a black urban population in South Africa (the Heart of Soweto Study): a cohort study. Lancet 2008; 371(9616): 915–22. DOI: 10.1016/S0140-6736(08)60417-1 18342686

[19] van Hagen IM, Baart S, Fong Soe Khioe R, Sliwa-Hahnle K, Taha N, Lelonek M, et al. Influence of socioeconomic factors on pregnancy outcome in women with structural heart disease. Heart 2018a; 104(9): 745–752. DOI: 10.1136/heartjnl-2017-311910 29092914

[20] van Hagen IM, Boersma E, Johnson MR, Thorne SA, Parsonage WA, Escribano Subias P, et al. Global cardiac risk assessment in the Registry Of Pregnancy And Cardiac disease: results of a registry from the European Society of Cardiology. Eur J Heart Fail 2016; 18(5): 523–33. DOI: 10.1002/ejhf.501 27006109

[21] van Hagen IM, Thorne SA, Taha N, Youssef G, Elnagar A, Gabriel H, et al. Pregnancy Outcomes in Women With Rheumatic Mitral Valve Disease: Results From the Registry of Pregnancy and Cardiac Disease. Circulation 2018b; 137(8): 806–16. DOI: 10.1161/CIRCULATIONAHA.117.032561 29459466

[22] Watkins DA, Johnson CO, Colquhoun SM, Karthikeyan G, Beaton A, Bukhman G, et al. Global, regional, and national burden of rheumatic heart disease, 1990–2015. N Engl J Med 2017; 377(8): 713–22. DOI: 10.1056/NEJMoa1603693 28834488

[23] Watkins DA, Sebitloane M, Engel ME, Mayosi BM. The burden of antenatal heart disease in South Africa: a systematic review. BMC Cardiovasc Disord 2012; 12: 23 DOI: 10.1186/1471-2261-12-23 22463484 PMC3340323

[24] World Health Organization. Global status report on noncommunicable diseases 2014 Geneva; 2014. 10.1161/STROKEAHA.115.008097 25873596

[25] WHO & The Parternship for Maternal, Newborn and Child Health. Maternal and child health: KENYA. 2011 Available from: http://www.who.int/pmnch/media/membernews/2011/20121216_kenyaparliament.pdf.

[26] WHO, UNICEF, UNFPA, World Bank Group and the United Nations Population Division. Trends in maternal mortality: 1990 to 2015 estimates by WHO, UNICEF, UNFPA, World Bank Group and the United Nations Population Division Geneva: WHO Press; 2015 [Available from: https://data.unicef.org/wp-content/uploads/2015/12/Trends-in-MMR-1990-2015_Full-report_243.pdf.

[27] Yego F, D’Este C, Byles J, Williams JS, Nyongesa P. Risk factors for maternal mortality in a tertiary hospital in Kenya: a case control study. BMC Pregnancy Childbirth 2014; 14: 38 DOI: 10.1186/1471-2393-14-38 24447854 PMC3904405

[28] Yego F, Stewart Williams J, Byles J, Nyongesa P, Aruasa W, D’Este C. A retrospective analysis of maternal and neonatal mortality at a teaching and referral hospital in Kenya. Reprod Health 2013; 10: 13 DOI: 10.1186/1742-4755-10-13 23421605 PMC3599365

[29] Zuhlke L, Engel ME, Karthikeyan G, Rangarajan S, Mackie P, Cupido B, et al. Characteristics, complications, and gaps in evidence-based interventions in rheumatic heart disease: the Global Rheumatic Heart Disease Registry (the REMEDY study). Eur Heart J 2015; 36(18): 1115–22a. 25425448 10.1093/eurheartj/ehu449 PMC4422972

[30] Zuhlke L, Karthikeyan G, Engel ME, Rangarajan S, Mackie P, Cupido-Katya Mauff B, et al. Clinical outcomes in 3343 children and adults with rheumatic heart disease from 14 low- and middle-income countries: Two-year follow-up of the Global Rheumatic Heart Disease Registry (the REMEDY Study). Circulation 2016; 134(19): 1456–66. DOI: 10.1161/CIRCULATIONAHA.116.024769 27702773

